# Impact of the COVID-19 Pandemic on Acute Admissions to a Secondary Referral Hospital in a Rural City in Japan: A Retrospective Study

**DOI:** 10.1017/dmp.2021.84

**Published:** 2021-03-25

**Authors:** Hiroki Sato, Hidekazu Kondo, Takahiro Oniki, Keiichi Uraisami, Hideyuki Watanabe, Seigi Koga, Masaaki Chinen, Hiroshi Sakino, Seiki Nobe, Naohiko Takahashi

**Affiliations:** 1 Department of Internal Medicine, Kunisaki City Hospital, Oita, Japan; 2 Department of Cardiology and Clinical Examination, Faculty of Medicine, Oita University, Oita, Japan

**Keywords:** COVID-19, respiratory diseases, nonpharmaceutical measures

During the coronavirus disease 2019 (COVID-19) pandemic, the fear of contracting COVID-19 and preventive measures may have influenced people’s health.^1^ For example, emergency department (ED) use has decreased.^1^ The influence of the COVID-19 pandemic on people’s health in low-incidence areas is unclear. Assessment of the influence of COVID-19 in low-incidence areas would contribute to understanding the relationship between the scale of the pandemic and its effect on people’s health.

The 208-bed Kunisaki City Hospital is a secondary level emergency hospital with 4,542 ED visits in 2019, providing in-hospital care for patients with moderately severe illness for the more than 27,000 residents of Kunisaki City in Oita Prefecture, Japan. Until June 30, 2020, no COVID-19 cases were reported in Kunisaki, and 60 cases were reported in Oita Prefecture.^2^ The Japanese government asked the public to refrain from attending mass gatherings or traveling to other prefectures but did not impose lockdown measures. This study aimed to determine the influence of the COVID-19 pandemic on acute hospital admissions in a low-incidence setting.

Data on acute admissions between January 2018 and June 2020 were collected retrospectively. An acute admission was defined as a physician deciding that a patient needed immediate admission because of their disease severity and treatment requirements. The mean number of acute admissions per month during the prepandemic (January 2018 to December 2019) and pandemic (January 2020 to June 2020) periods were compared according to the International Classification of Diseases (ICD-10) disease categories. The Medical Ethics Committee of the Kunisaki City Hospital approved this study, which conformed to the Helsinki Declaration.

The number of acute admissions related to respiratory diseases decreased significantly during the pandemic period, while the number of acute admissions related to cardiovascular diseases increased significantly (Table 1). There was no significant change in infectious or noninfectious respiratory disease-related admissions, and no significant changes in the number of admissions in other disease categories.

**Table 1.** Mean number of acute admissions per month

	Prepandemic^a^	Pandemic^b^	*P*-Value
Respiratory diseases	24.8 ± 6.2	18.8 ± 5.1	0.037*
	(n=596)	(n=113)	
Infectious	18.9 ± 6.2	14.7 ± 4.3	0.126
	(n=454)	(n=88)	
Noninfectious	5.9 ± 2.4	4.2 ± 2.3	0.121
	(*n* = 142)	(*n* = 25)	
Cardiovascular diseases	22.1 ± 4.9	24.7 ± 1.5	0.037*
	(*n* = 530)	(*n* = 148)	

Note: There were no significant differences for other disease categories (data not shown).

a Prepandemic period: January 2018 to December 2019.

b Pandemic period: January 2020 to June 2020.

* Statistically significant.

A previous study suggested that social distancing measures and behavioral changes, including personal hygiene measures, and fear of COVID-19, might contribute to a reduction in the incidence of infectious respiratory diseases.^3^ This study showed a significant reduction of acute admissions for respiratory diseases; however, the incidence of infectious respiratory diseases did not change. This might be because of the small sample size.

Our results, which show a significant increase in acute cardiovascular disease admissions during the pandemic period, contrast with those of previous studies.^4,5^ The scale of pandemic within the country; behavioral changes due to fear of COVID-19; and the health-care system, including access to emergency medical services, might have contributed to the increase. Multicenter studies of cardiovascular disease patient characteristics during the prepandemic and pandemic periods in areas with varying COVID-19 incidence rates may reveal the reasons for the increase of acute cardiovascular disease admissions to our hospital during the COVID-19 pandemic period and reasons for the difference between our results and those of previous studies.
